# Genomic landscape of HIV-1 drug resistance in Africa: a comprehensive analysis of mutation trends, subtype diversity, and therapeutic implications (1990–2024)

**DOI:** 10.1093/bib/bbaf631.014

**Published:** 2025-12-12

**Authors:** Halleluyah Oludele, Abdul Latif Koney Shardow, Maame Apaflo, Jonas Ibekwe, Phazha Baeti, Julien Nguinkal, Olaitan Awe

**Affiliations:** University of Ibadan, Nigeria; Kumasi Centre for Collaborative Research, Ghana; Noguchi Memorial Institute, Ghana; University of Ibadan, Nigeria; University of Botswana; Bernhard-Nocht-Institut, Germany; African Society for Bioinformatics and Computational Biology

## Abstract

**Background:**

Drug resistance-associated mutations (DRMs) in HIV-1 remain critical barriers to antiretroviral therapy success in Africa. We analyzed 105,970 HIV-1 pol sequences spanning 30+ years across 40 African countries to quantify DRM evolution.

**Methods:**

HIV-1 pol sequences (1990–2024) from Los Alamos database were analyzed. From 217,000 entries, 105,970 sequences underwent resistance profiling using SierraPy. Analysis included temporal assessments, subtype diversity, mutation quantification, and resistance clustering using ARIMA modeling.

**Results:**

DRMs detected in >34% of sequences. NNRTI mutations (K103N, Y181C) were most prominent, peaking 2000–2016. Strong cross-resistance: Efavirenz-Nevirapine (r = 0.98), NRTI combinations (r > 0.90). INSTI resistance increased post-2016 with EVG-RAL overlap (r = 0.96). Predictive modeling showed persistent resistance across all classes: NNRTIs >80%, heterogeneous NRTI patterns (Zidovudine 97%, Tenofovir favorable).

**Conclusion:**

High DRM prevalence (>34%), strong cross-resistance, and emerging INSTI resistance underscore needs for genotype-guided therapy, resistance testing, and tailored ART policies across Africa.

**References:**

1. Hamers R.L. et al. ‘HIV-1 drug resistance in antiretroviral-naive individuals in sub-Saharan Africa after rollout of antiretroviral therapy: a multicentre observational study.’ Lancet Infectious Diseases 2011;11:750–759.

2. Gupta R.K. et al. ‘HIV-1 drug resistance before initiation or re-initiation of first-line antiretroviral therapy in low-income and middle-income countries.’ Lancet Infectious Diseases 2018;18:346–355.

